# Effects of six codend meshes on the size selection of juvenile white croaker (*Pennahia argentata*) in demersal trawl fishery of the South China Sea

**DOI:** 10.1371/journal.pone.0253723

**Published:** 2021-07-16

**Authors:** Bingzhong Yang, Bent Herrmann, Lei Yan, Jie Li, Teng Wang

**Affiliations:** 1 South China Sea Fisheries Research Institute, Chinese Academy of Fishery Sciences, Guangzhou, China; 2 Key Laboratory of Open-Sea Fishery Development, Ministry of Agriculture and Rural Affairs, Guangzhou, China; 3 SINTEF Ocean, Fishing Gear Technology, Hirtshals, Denmark; 4 University of Tromsø, Breivika, Tromsø, Norway; 5 DTU Aqua, Technical University of Denmark, Hirtshals, Denmark; Hellenic Center for Marine Research, GREECE

## Abstract

White croaker (*Pennahia argentata*) is a commercially important but overexploited species that is often caught in trawl fishery of the South China Sea (SCS). The codend size selectivity for this species in the local commercial trawl fishery is of concern when considering the established minimum landing size (MLS). This study investigated the size selectivity of white croaker for six different diamond-mesh codends with mesh size from 25 to 54 mm. We paid special attention to two codends, made with meshes of 25 and 40 mm in size, which are currently used according to the regulations established in the SCS. The results demonstrated that the legal codends do not perform satisfactorily in the fishing grounds where juvenile white croaker is relatively abundant. This is because at a length similar to the minimum landing size of the species (MLS = 15.0 cm), all white croaker were retained, and the estimated discard ratio was >97% in both cases of legal codends. Our study showed that by increasing the mesh size, the size selection of tested codends could be improved for white croaker, and the retention rates for juvenile fish would decrease. However, none codend was proved efficient to release undersized white croaker suggesting that other gear design changes may be necessary.

## Introduction

White croaker *(Pennahia argentata*, (Houttuym, 1782)) is an important demersal fish species for trawl fishery, widely distributed in the coastal waters of China, Japan and Korea [1]. In 2018, the total production of white croaker was 95 889 t, of which 23 284 t (accounted for 24.28%) was from the Chinese domestic marine fisheries in the South China Sea (SCS) [2]. However, the stock of white croaker has been overexploited and even declined, especially in the SCS [3–5]. Many factors have contributed to the exhaustion of this resource, among them, the capture of high quantities of juvenile fish might be proved to be the most important one. For instance, Zhang et al. [6] recently reported that about 42.86% of the total amounts of white croaker caught by all types of fishing gears were juveniles. Additionally, an earlier survey conducted by Yang et al. [7] revealed that 100% of the white croaker caught by a shrimp trawl was undersized.

The poor size selection of trawl, especially in the codend, is believed to be the main reason for the important juvenile catch by the Chinese fishing fleet. This is related to the small diamond mesh size used in the trawl codend, often close to 25 mm or illegally smaller. Despite the fact that China has established a minimum mesh size (MMS) of 40 mm for the codend of trawls targeting fish and 25 mm for those targeting shrimps, the enforcement and effectiveness of these regulations are widely doubted and criticized [6, 8, 9]. Low compliance of these regulations can be due to many factors. One of the most important causes is the gap in knowledge on the selection of the fishing gears. Specifically, the size selection of the codends with minimum mesh sizes in the SCS has not been tested and quantified for the economically important species, such as white croaker. Understanding the size selection of fishing gear for the target species, especially for the juvenile fish, is the basis for decision-making of fishery management to protect and recover of a specific fishery resource. In Chinese domestic marine fishery, the minimum landing size (MLS) for white croaker is 15.0 cm in total length. However, at present there is no literature and experiments regarding how the size selection of codends with different mesh sizes, including the two legal ones, 25 and 40 mm, would match the MLS of this species. Furthermore, there is no study focused on the size selection of juvenile white croaker in the demersal trawl fishery of the SCS. So, one of the societal concerns is whether the established MMS regulations are effective enough to protect juvenile fish of the specific species.

Apart from the estimation of the size selection of the fishing gears, which is independent of the fished population, it is also relevant to quantify the exploitation pattern for the fish population available in the studied fishing grounds. The estimation of the exploitation pattern indicators can provide quantitative information about the suitability of fishing gears for the specific fishing situation in terms of the capture pattern and efficiency [10, 11]. Catch efficiency can provide information to fishermen about how the retention of fish above the MLS can be impacted if the codends with legal mesh sizes were applied.

To address the issues mentioned above, the main objective of this study was to fill the knowledge gap about the size selection of different codends for white croaker in demersal trawl fishery of the SCS. By testing and comparing selective properties of six diamond-mesh codends, with mesh size of 25, 30, 35, 40, 45 and 54 mm, respectively, for white croaker we focused on the following research questions:

1. To what extent is the size selection of codends with the legal mesh size, 25 mm and 40 mm, satisfactory for white croaker, considering the MLS regulation?
2. Can the size selection and exploitation pattern of codends for white croaker be different by simply changing the mesh sizes?
3. If there are differences in the size selection and exploitation pattern between codends with different mesh sizes, are they length-dependent?

## Materials and methods

### Ethics statement

This study did not involve any endangered or protected species. Experimental fishing was conducted onboard a commercial vessel in accordance with the fishing permit granted by the Ministry of Agriculture and Rural Affairs of China. No other authorization or ethics board approval was required to carry out this study. Information on animal welfare and steps to ameliorate suffering and methods of sacrifice is not applicable, as the animals were not exposed to any additional stress other than that involved in commercial fishing practices.

### Study area and data collection

Data were collected in sea trials performed onboard a commercial trawler, named “Guibeiyu 96899” (280 kW, 38 m), in October 2019. To make sure that all fishing conditions and procedures were identical to the commercial fishing, the fishing experiments were conducted in the Beibu Gulf of the northern SCS (N20°50’-21°11’, E109°06’-109°31’), which is a traditional fishing ground with a depth range of about 12 to 24 m for the commercial fleet. In order to avoid the potential effects of different fishing conditions on the selectivity of the tested codends, towing speed and duration for each haul were kept relatively constant at commercial level, about 3.5 knots and 2 hours, respectively. During the sea trials, the experimental fishing was conducted day and night continually, which is typical for the commercial fishery.

### Fishing gear and experimental set-up

**Fig 1:**
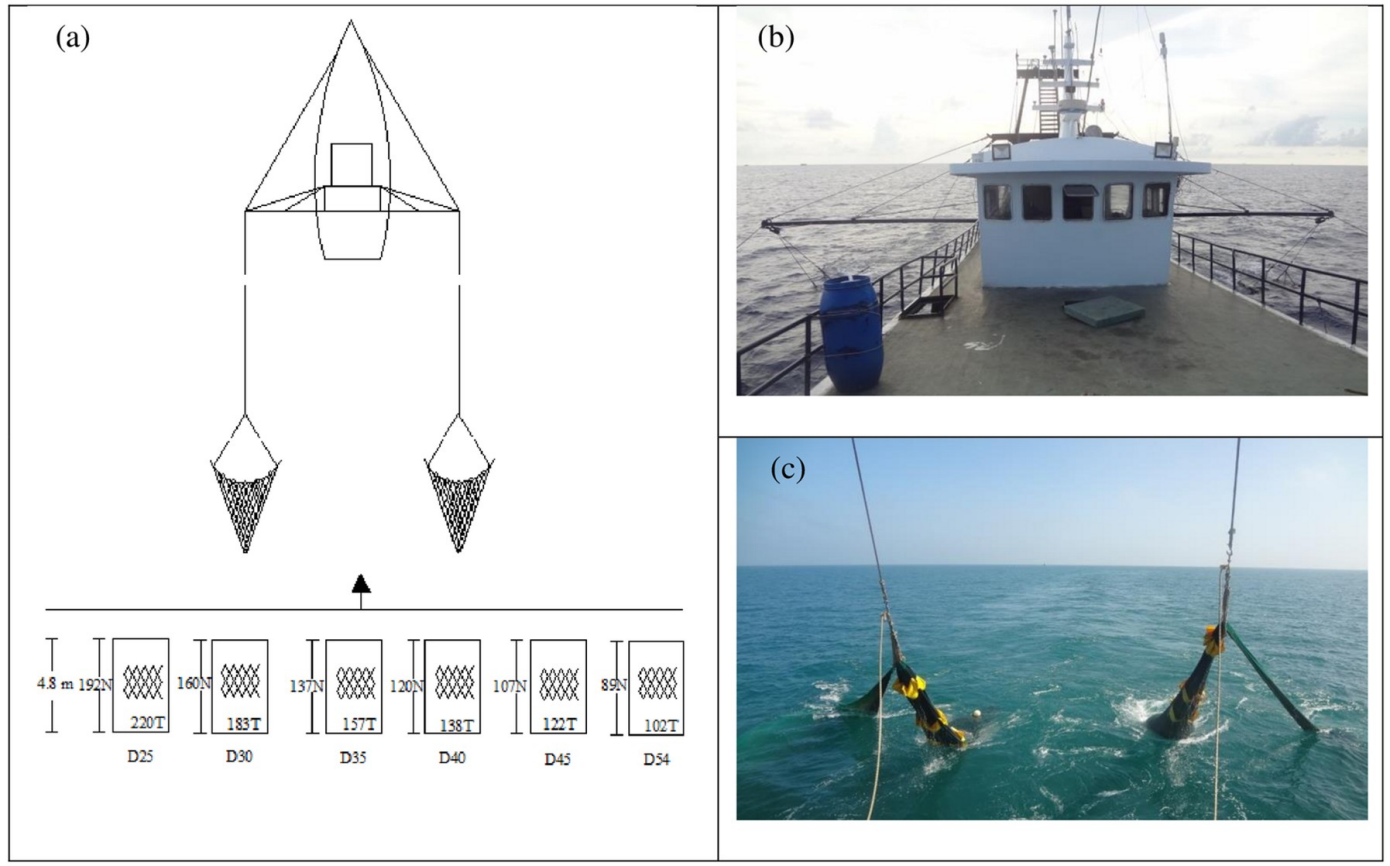
Schematic view of the fishing gear configuration tested in the experiments. (a): the fishing vessel and specification of the tested codends, (b): the vessel towing two trawls simultaneously, and (c): the haul-back process of the tested codends.

Since the commercial fishing vessels are equipped with a double-rigged trawl system, in which two trawls could be hauled and retrieved by the same vessel simultaneously and separately (Fig 1), for the present study, we used the trawl system of the hired commercial vessel with the same gear set-up except for the part of the codend. The trawls located in the port and starboard sides were identical, both in material and design characteristics. They all had a fishing circumference of 860 meshes, with a mesh size of 45 mm, and a total stretched length of ~33 m. The mesh size was 45 mm in the wings and 30 mm in the extension. Two identical sets of trawl doors (each 250 kg and 1.6 m^2^), made of wood and steel, were used to tow each trawl. During normal fishing, the headline height of the fishing gear was about 1.5 m, and distance between doors was about 15 m.

Six diamond-mesh codends with different inside stretched mesh sizes, 25, 30, 35, 40, 45 and 54 mm, respectively, were designed and tested using the covered codend method. Based on their mesh sizes, these codends were referred to as D25, D30, D35, D40, D45 and D54, respectively. All the tested codends were designed taking into consideration the design of the commercial codend, which had a circumference of 220 meshes (25 mm in size) and a total stretched length of 4.8 m. Apart from the mesh size, the tested codends were identical to the commercial one in twine material (knotted green polyethylene netting), diameter, and stretched dimension. To neutralize the potential effect of the codend circumference to the size selection, the mesh number around the codend reduced as the mesh size increased to obtain a similar codend circumference in all cases. The dimension of the cover was 1.5 times larger than the tested codend, following the recommendation of Wileman et al. [12]. Measurements of mesh sizes (mesh openings) were conducted according to the protocol described in Wileman et al. [12]. Detailed information about the codends and cover used is listed in Table 1 and Fig 1. In order to avoid the masking effect, flexible kites made of waterproof canvas [13, 14] were equipped in the front, middle and back part (potential catch accumulation zone of codend) of the cover, 12 kites in total. Before the formal experiments, two underwater video recording systems, comprised of GoPro HERO4 BLACK Edition and the supported frame, were used to check whether the cover would mask the tested codend.

**Table 1:** Overview of specifications of the tested codends.

codend	mesh opening±SD (mm)	twine diameter±SD (mm)	mesh number in circumference	mesh number in length
D25	25.91±1.05	1.40±0.36	220	192
D30	29.74±0.70	1.24±0.11	183	160
D35	35.70±1.14	1.31±0.10	157	137
D40	40.40±0.85	1.36±0.17	138	120
D45	44.28±0.66	1.24±0.09	122	107
D54	54.54±0.86	1.26±0.09	102	89
cover	12.51±0.78	1.18±0.10	550	480

SD represents standard errors.

As the fishing vessel was able to haul two trawls simultaneously, we arranged three pairwise experimental fishing tests: D25 vs. D30, D35 vs. D40 and D45 vs. D54, one at a time, to explore how would the mesh size impact the selection of the codends. To remove the potential bias, we made sure that fishing procedure of the two trawls was conducted simultaneously. The common fishing conditions for all hauls were nearly the same in terms of the tow duration and water depth (detailed information are listed in the S1 Table). After the haul-back process, catches from each compartment, codend and cover, were processed separately for each tested codend. All catches of white croaker were sorted, subsampled (if needed), and frozen for length measurement in the laboratory. Total length of sampled catches was measured to the nearest millimetre, providing the number of individuals in the catch of each compartment, cover and codend, for further analysis on the size selectivity.

### Selectivity estimation

Despite the fact that there were two codends simultaneously tested each time in our experiments, for each specific codend fish were either retained by the cover or the codend. We analyzed the fishing data for each codend separately as binominal distribution. The proportion (probability) of a given fish with length *l* retained by each specific codend in haul *j* was expressed as *r*_*j*_*(l)*. The value of *r*_*j*_*(l)* can be calculated by the number of fish of length *l* retained by the codend and the total number of fish of length *l* entering the codend. For the same codend, however, the value of *r*_*j*_*(l)* would be expected to vary between hauls [15]. In the present study, our main interest was the averaged over hauls length-dependent values of *r (l)*, because this would provide about outcomes for the size selection process of using a specific codend in the fishery. Thus, it was assumed that size selective performance of the tested codend in the experiment was representative of how the codend would perform in a commercial fishery [16–18]. As a result, we used *r*_*av*_*(l)* to represent the average size selection estimated by pooling data from all hauls conducted with each specific codend [19]. Different parametric models were tested and compared for *r*_*av*_*(l)*, where *v* is a vector consisting of the parameters of each model. The purpose of this analysis is to estimate the values of parameter *v* that make experimental data (averaged over hauls) most likely to be observed, by assuming that the model is able to describe the data sufficiently well. Thus, Eq (1) was minimized the respect to parameter *v*, which was equivalent to maximizing the likelihood for the observed data in form of the length-dependent number of fish caught by the codend (*nR*_*jl*_) versus those escaping to the cover (*nE*_*jl*_):

$$-\sum_{\text{j}=1}^{\text{m}}\sum_{l}\left\{\frac{nR_{jl}}{qR_{j}}\times \text{ln}\left(r_{av}\left(l,v\right)\right)+\frac{nE_{jl}}{qE_{j}}\times \text{ln}\left(1.0-r_{av}\left(l,v\right)\right)\right\}$$ (1)

where the outer summation is over the *m* hauls conducted, while the inner summation is over length classes *l*; *qR*_*j*_ and *qE*_*j*_ are the sub-sampling factors for the fraction of the fish length measured in the codend and cover, respectively.

Four basic models, Logit, Probit, Gompertz and Richards, were chosen as candidates to describe *r*_*av*_*(l)* [12]. The first three models can be fully presented by two selection parameters L50 (length of fish with 50% probability of being retained) and SR (= L75-L25). For the Richards model, an additional parameter, 1/δ, is required to describe the asymmetry of the curve. Detailed information about these models can be found in Wileman et al. [12].

The selection of the best model among the candidates was conducted by their AIC values. The model with the lowest AIC values is selected as the best one [20]. The ability of a model to describe the data sufficiently well can be evaluated by inspecting the corresponding *p*-value, which expresses the likelihood of obtaining at least as big a discrepancy between the fitted model and the observed experimental data as would be expected by coincidence. For the fitted model to be a candidate to model the size selection data, the *p*-value should not be less than 0.05 [12]. In case of a poor statistical fit (*p*-value < 0.05), the residuals would be inspected to determine whether the result was due to structural problems when modelling the experimental data using the different selection curves or if it was due to overdispersion in the data [12].

After the specific size selection model was identified for a given codend, bootstrapping was applied to estimate the confidence limits for the average size selection. We applied the software tool SELNET [19] for the size selection analysis and used the double bootstrap method implemented in the tool to obtain confidence limits for the size selection curve and the corresponding parameters. This bootstrapping approach, which takes both within-haul and between-haul variation into account, is identical to the one described in Millar [16]. A “pooled” set of data was analyzed using the identified selection model, then 1000 bootstrap repetitions were conducted to estimate the Efron percentile 95% confidence limits for the selection curve and its parameters [19].

### Estimation of exploitation pattern indicators

In order to test and compare how these codends with different mesh sizes perform under the same fishery population of white croaker, a specific scenario of population, *nPop*_*l*_, was generated by pooling cover and codend data over all hauls conducted with all codends[21, 22]. We assumed that fishing population (*nPop*_*l*_) was the same for all codends. This was supported by the fact that i) the hauls conducted represent the fishing ground of the fleet and ii) in all codends, the size structure of the total amount of fish entering each specific codend represent the juvenile part of the population of the species. Applying the size selection curves predicted in the previous section, four exploitation pattern indicators, *nP-*, *nP+*, *nRatio*, and *dnRatio* (Eq 2), were calculated for each codend using the MLS of white croaker in the studied area. These indicators directly depend on the size structure of the fish population encountered during the experimental fishing, and can provide additional information for the evaluation of the exploitation pattern of the tested codends.

$$\begin{array}{c} nP-=100\times \frac{\sum_{l<MLS}\left\{r_{codend}\left(l\right)\times nPop_{l}\}\right.}{\sum_{l<MLS}\left\{nPop_{l}\}\right.}\\ nP+=100\times \frac{\sum_{l\geq MLS}\left\{r_{codend}\left(l\right)\times nPop_{l}\}\right.}{\sum_{l\geq MLS}\left\{nPop_{l}\}\right.}\\ nRatio=\frac{\sum_{l<MLS}\left\{r_{codend}\left(l\right)\times nPop_{l}\}\right.}{\sum_{l\geq MLS}\left\{r_{codend}\left(l\right)\times nPop_{l}\}\right.}\\ dnRatio=100\times \frac{\sum_{l<MLS}\left\{r_{codend}\left(l\right)\times nPop_{l}\}\right.}{\sum_{l}\left\{r_{codend}\left(l\right)\times nPop_{l}\}\right.} \end{array}$$ (2)

where *r*_*codend(l)*_ is the size selection curve obtained for the specific codend, while *nPop*_*l*_ represents the size structure of white croaker entering the codends in terms of individuals of length class *l*. *nP*- and *nP*+ is the percentage retained fish below and above the MLS, respectively, taking into account the size structure of the population encountered during the experimental fishing. It would be preferable to have an *nP*- value close to 0 and an *nP*+ value close to 100. *nRatio* is the landing ratio between the retained fish below and above the MLS. The *dnRatio* is the percentage of fish individuals below the MLS retained by the codend. Both *nRatio* and *dnRatio* should be as low as possible. The double bootstrapping approach was applied to estimate the Efron percentile 95% confidence interval (CI) for the indicator values, taking both within-haul and between-haul variation into consideration [19].

### Difference in size selection

Since the fishing conditions were common for all tested codends in the same fishing ground onboard the same commercial vessel, it is possible to compare the size selectivity of different codends under the same circumstance. In order to infer the effect of increasing mesh size on size selection of different codends, the difference Δ*r*(*l*) was estimated by:

$$\Delta r(l)=r_{B}(l)–r_{A}(l)$$ (3)

where *r*_*A*_ (*l*) is the value of selection curve for codend A (with small mesh size), and *r*_*B*_ (*l*) represents the value of selection curve for codend B (with relatively larger mesh size). Efron 95% percentile confidence limits for Δ*r*(*l*) could be obtained based on two bootstrap populations of results for both *r*_*A*_ (*l*) and *r*_*B*_ (*l*). As they were obtained independently, a new bootstrap population of results was created for Δ*r*(*l*) by:

$$\Delta r{\left(l\right)}_{i}=r_{B}{\left(l\right)}_{i}-r_{A}{\left(l\right)}_{i}\;i\in [1\ldots 1000]$$ (4)

where *i* is the bootstrap repetition index. As the bootstrap re-sampling was random and independent for the two groups of results, it is valid to generate the bootstrap population of results for the difference based on Eq (4), by using the two independently generated bootstrap files [23, 24].

## Results

### Experimental data

**Fig 2:**
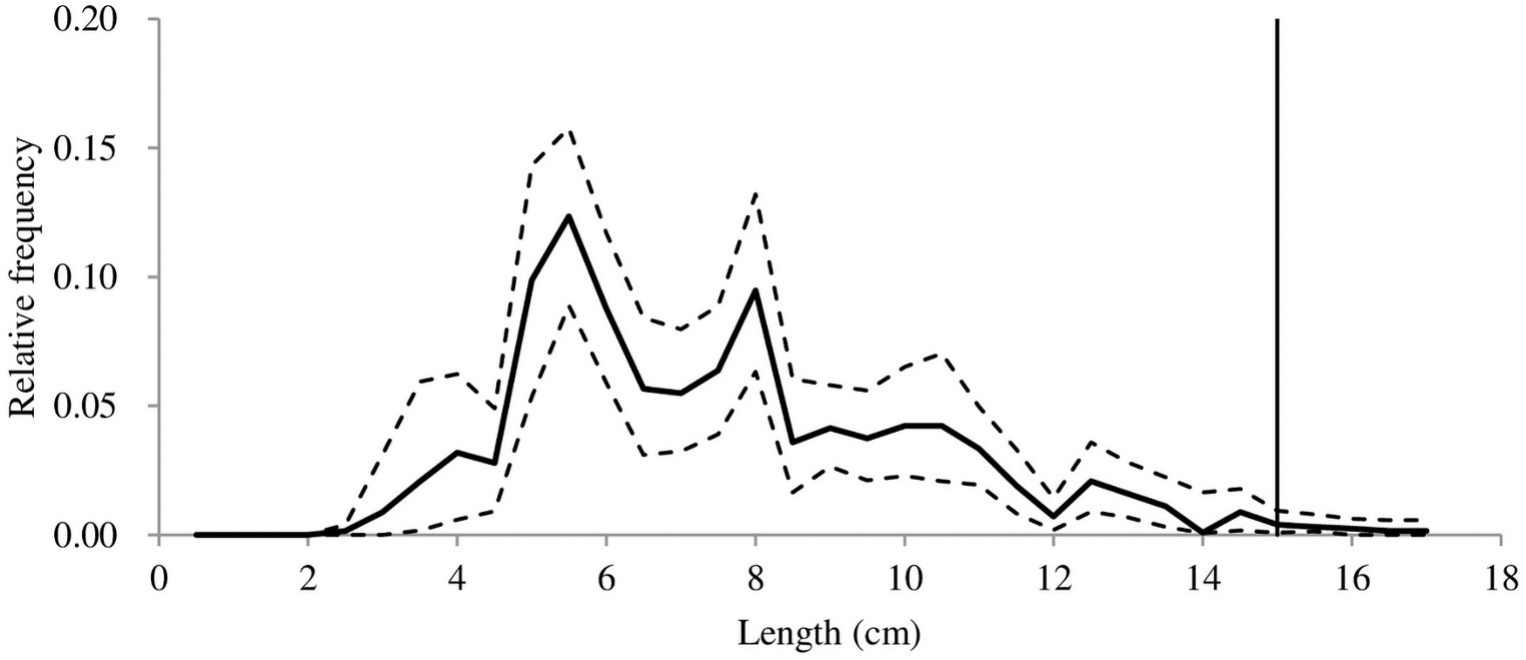
Estimated average population from all hauls during the sea trials. Stipple lines show the 95% Efron confidence intervals, and the vertical line represents the MLS (minimum landing size) of white croaker.

**Fig 3:**
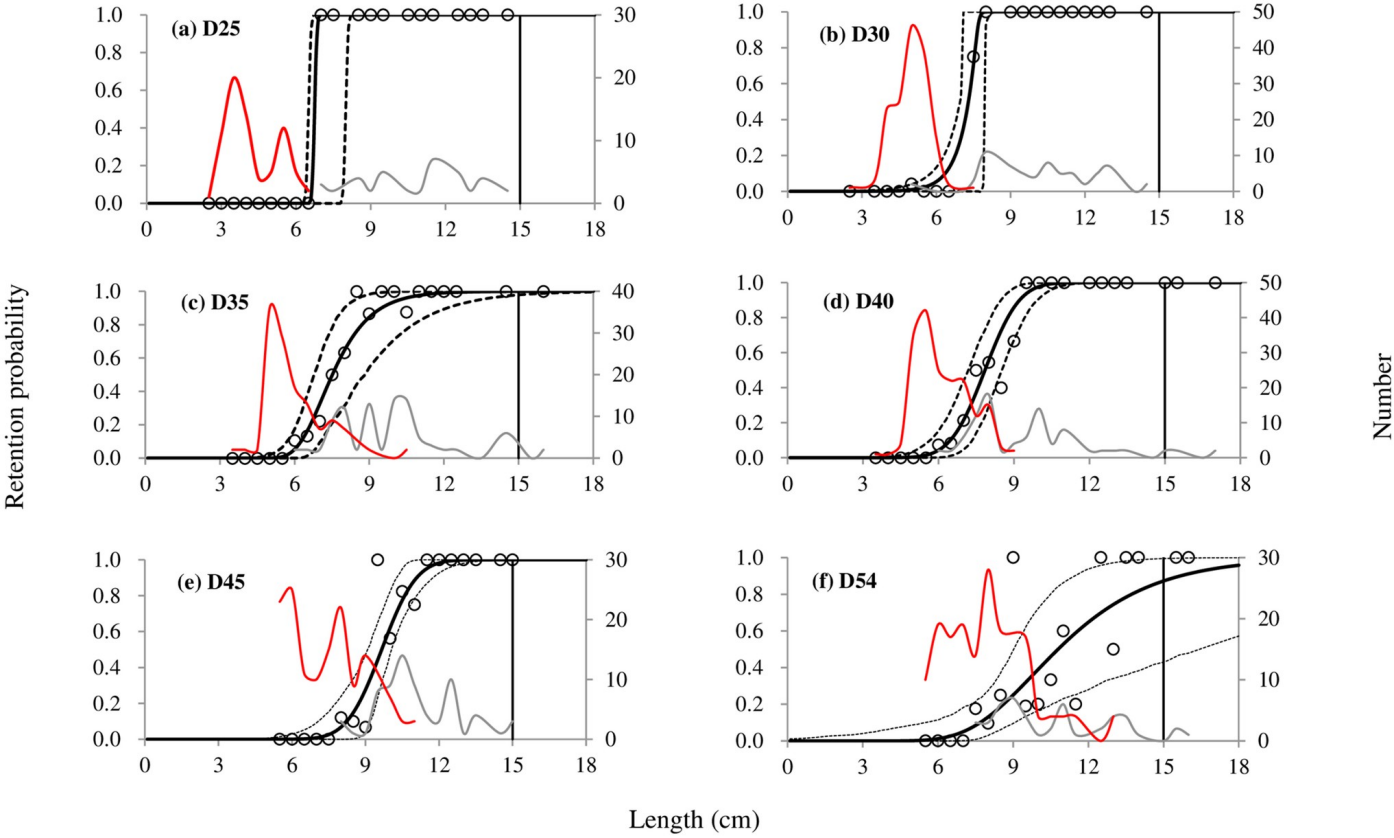
Experimental catch proportion and selectivity curves. Circle marks represent experimental catch proportion. Red curves represent the size distribution of fish caught by the cover, grey curves represent the one caught by the tested codend. Stippled curves describe the 95% confidence intervals for the fitted selection curves. Vertical lines represent the MLS (minimum landing size).

In total, 45 valid hauls were conducted, seven hauls for the D25, D45 and D54, and eight hauls for the D30, D35 and D40 codend, respectively (Table 2). The towing duration was mainly 2 h, with a range between118 and 156 min. White croaker was present at all hauls and the subsampling ratio varied from 0.20 to 1.00. A total of 677 white croaker was length measured. The total length of the measured individuals ranged from 2.5 to 16.8 cm, and most of them were undersized fish when compared to the MLS value (15.0 cm) representing in all cases the juvenile part of the population of the studied species (Figs 2 and 3).

**Table 2:** Overview of catch data in the sea trial.

Data specification	Codend					
	D25	D30	D35	D40	D45	D54
No. of hauls	7	8	8	8	7	7
Duration range (min)	118–156	118–156	128–153	128–153	120–128	120–128
No. of individuals in codend	18	29	40	46	55	36
No. of individuals in cover	74	146	71	82	42	38
Sub-sampling factor in codend	0.33–0.50	0.33–0.50	0.33–0.50	0.50–0.50	0.50–1.00	0.50–1.00
Sub-sampling factor in cover	1.00–1.00	1.00–1.00	0.33–1.00	0.25–1.00	0.25–0.33	0.20–0.33
Length range (cm)	2.7–14.3	2.5–14.4	3.6–16.2	3.4–16.8	5.5–15.0	5.7–16.0

No. of individuals in codend and cover represents the number of measured individuals.

### Size selectivity

In order to choose the best model for each of the tested codend, four size selection models were fitted to the pooled data. By comparing their AIC values (Table 3), the Probit model was considered to be the best one for the D25, D40 and D45 codend, respectively; the Gompertz model was the best for the D35 and D54 codend, respectively. For the D30 codend, we chose the Richards model. Using these models, we obtained selection parameters and fit statistics for the tested codends (Table 4). The results of the fit statistics showed that *p*-values were larger than 0.05, except for the D54 codend. However, as the selection curve represents the main trend of the experimental data well (Fig 3(f)), we considered that this low *p*-value was due to overdispersion in the data.

**Table 3:** Akaike’s information criterion (AIC) for each model of the tested codends.

codend	Models			
	Logit	Probit	Gompertz	Richards
D25	4.04	**4.00**	4.02	6.00
D30	36.30	38.93	47.47	**34.29**
D35	123.58	124.23	**119.61**	121.74
D40	165.20	**163.27**	164.82	166.19
D45	110.36	**109.71**	110.53	111.83
D54	171.06	169.46	**167.37**	169.86

Selected model in bold.

**Table 4:** Selectivity parameters and fit statistics obtained from the selected models for the tested codends.

codend	model	L50 (cm)	SR (cm)	δ	*p*-value	deviance	DOF
D25	Probit	6.75 (6.50–8.00)	0.10 (0.10–0.10)		1.0000	0.00	19
D30	Richards	7.30 (6.95–7.93)	0.64 (0.01–0.97)	0.10 (0.10–0.62)	0.9947	5.75	17
D35	Gompertz	7.52 (6.78–8.84)	1.65 (0.84–2.87)		0.9984	5.78	19
D40	Probit	7.85 (7.18–8.52)	1.45 (0.95–1.88)		0.9989	6.52	21
D45	Probit	9.66 (9.09–10.06)	1.50 (0.62–2.31)		0.4498	17.07	17
D54	Gompertz	10.79 (9.03–16.44)	4.07 (1.86–13.68)		0.0356	28.88	17

In general, the size selection of the tested codends for white croaker was relatively poor when comparing the selective parameters with the MLS (15.0 cm). The retention probability of codends for white croaker with length similar to the MLS was high, 87.23% (CI: 42.96%-99.46%) for the D54 codend, and nearly 100% for the rest codends (Fig 3). The mean values of L50 increased as the mesh size of codends enlarged, from 6.75 cm for the D25 codend to 10.79 cm for the D54 codend. The differences of L50 between codends with mesh sizes less than 45 were not significant, as their confidence intervals overlapped. The D45 and D54 codend would result in significant larger L50 than those codends with smaller mesh sizes. Based on their 95% CI, the differences of L50 between the D45 and D54 codend were statistically insignificant. Concerning to SR, the D25 codend showed the lowest value (0.10) and statistically significant difference when compared with other codends (except the D30).

### Exploitation pattern indicators

The exploitation pattern indicators showed that the retention of undersized fish (*nP-*) reduced as the mesh size of codend increased, from 53.67% for the D25 codend to 16.76% for the D54 codend (Table 5). The D45 codend would have significantly lower *nP-* than the D25 and D30 codend, whereas the D54 codend significantly reduced *nP-* comparing with the D25, D30 and D40 codend, respectively. All codends had high retention for fish above the MLS, as *nP+* was as large as 90% and even above and the CIs were narrow except the D54 codend. *nRatio* reduced as the mesh size of codends increased, from 41.56 for the D25 codend to 14.42 for the D54 codend, but no significant difference was detected. High discard ratios were obtained for all codends, as *dnRatio* was larger than 93%, showing a decreasing trend but no significant difference was found.

**Table 5:** The exploitation pattern indicators obtained for the tested codends.

codend	*nP*_*-*_ (%)	*nP*_*+*_ (%)	*nRatio*	*dnRatio* (%)
D25	53.67 (36.78–65.39)	100.00 (100.00–100.00)	41.56 (19.13–105.83)	97.65 (95.03–99.06)
D30	47.15 (36.15–60.05)	100.00 (100.00–100.00)	36.52 (17.03–97.79)	97.33 (94.45–98.99)
D35	42.26 (27.73–55.74)	99.97 (98.38–100.00)	32.73 (14.92–90.60)	97.04 (93.72–98.91)
D40	40.25 (29.35–52.93)	100.00 (100.00–100.00)	31.17 (14.38–82.34)	96.89 (93.50–98.80)
D45	21.58 (14.89–31.53)	100.00 (99.98–100.00)	16.71 (7.72–46.07)	94.35 (88.53–97.88)
D54	16.76 (8.87–29.19)	90.00 (46.82–99.65)	14.42 (6.61–45.35)	93.52 (86.86–97.84)

### Differences in size selection

**Fig 4:**
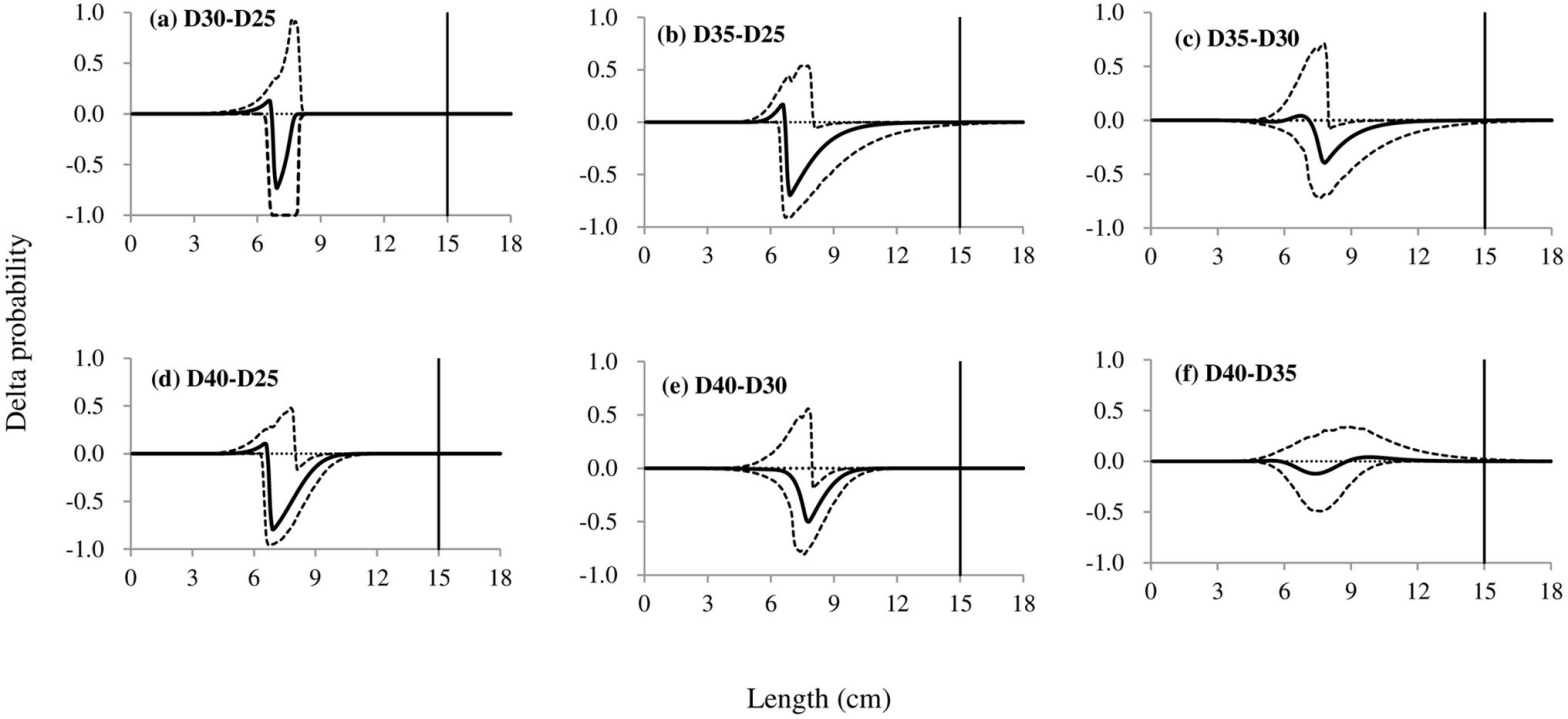
Delta selectivity curves from comparisons of four codends, the D25, D30, D35 and D40 codend. The solid black curves represent the differences of selectivity for each comparison, and the stippled curves represent the 95% confidence intervals. Vertical lines represent the MLS (minimum landing size).

**Fig 5:**
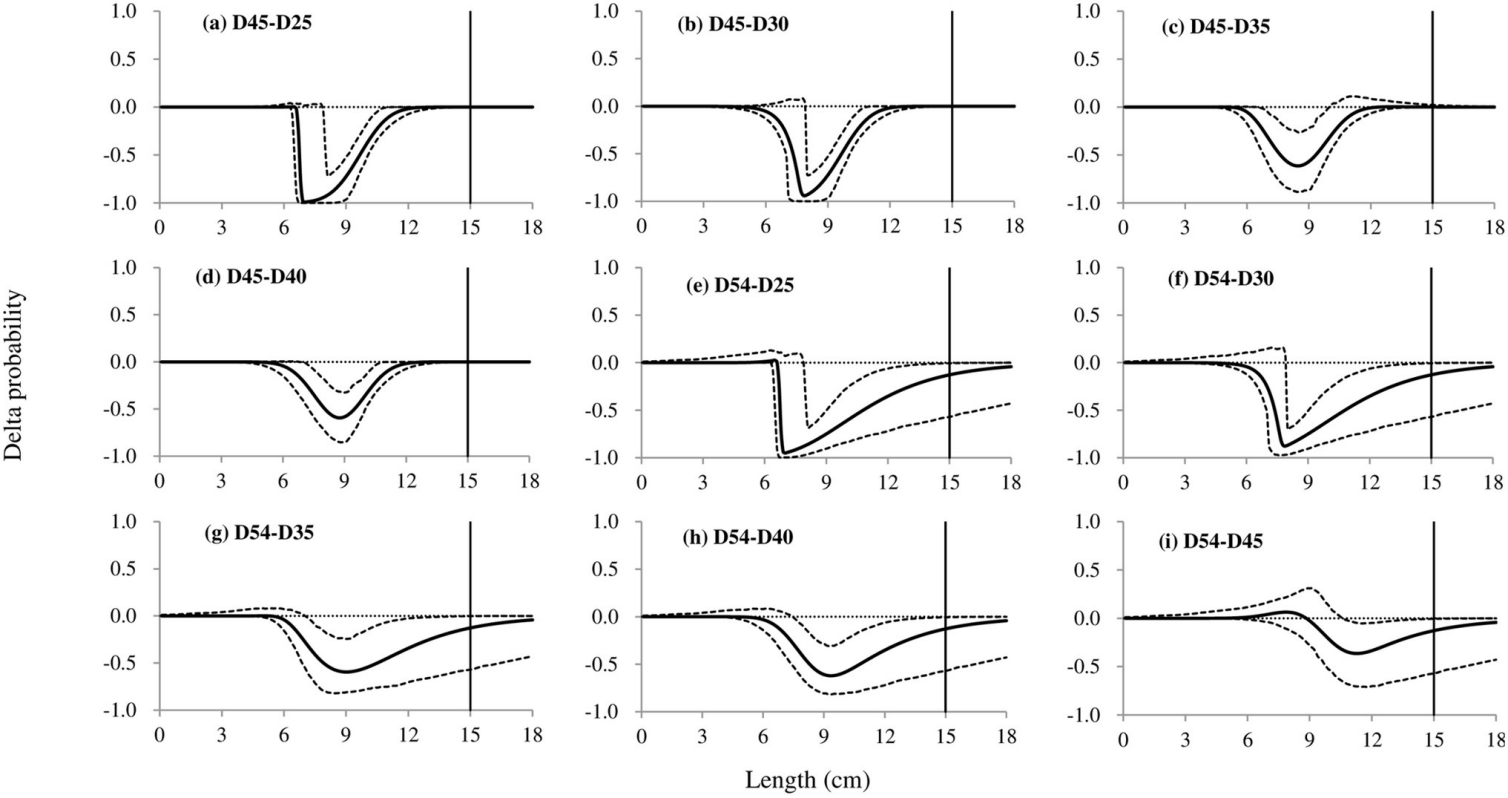
Delta selectivity curves from comparisons of six codends, the D25, D30, D35, D40, D45 and D54 codend. The solid black curves represent the differences of selectivity for each comparison, and the stippled curves represent the 95% confidence intervals. Vertical lines represent the MLS (minimum landing size).

The comparison in size selection between codends with different mesh sizes for white croaker showed that increasing the mesh sizes would reduce retention properties for some specific length ranges. The bigger the difference between the mesh sizes presented, the larger reduction obtained. Among these differences, only the differences between the D30 and D25 codend and between the D40 and D35 codend were not statistically significant (Fig 4(a) and 4(f)). Compared with the D25 codend, the D35 codend significantly reduced the retention rate of fish with length larger than 8 cm (Fig 4(b)). The retention properties of the D35 codend were significantly smaller than those of the D30 codend for individuals with length above 7.8 cm (Fig 4(c)). For the fish with length range of 8.1–12.5 cm, the D40 codend would have lower retention rate than the D25 codend; while substituting the D30 codend with the D40 codend would have a similar trend at the retention rate (Fig 4(d) and 4(e)). Using the D45 codend would obtain significant reduction for individuals with lengths ranged in 7.7–12.7 cm, compared with the D25 and D30 codend, respectively (Fig 5(a) and 5(b)). For fish with length ranged in 6.5–9.9 cm, the D45 codend significantly reduced the retention rates than the D35 codend, while a similar effect was obtained for the D45 codend compared to the D40 codend for fish with lengths of 6.9–11.1 cm (Fig 5(c) and 5(d)). Applying the D54 codend would result in significant reduction at retention rates than all the other codends for individuals with specific length ranges. Comparing with the D25, D30, D35 and D40 codend, the effect was manifest for fish with length above 7.7 cm (Fig 5(e)–5(h)), while with the D45 codend a bigger length would be required, larger than 11.7 cm (Fig 5(i)).

## Discussion

Quantification of the size selection of trawl codend is essential for the optimization of gear design and the technical management regulation of a fishery. In the present study, we investigated the size selection of six diamond mesh codends, with a wide range of mesh sizes from 25 mm to 54 mm, for white croaker for the first time in the SCS under a commercial fishing condition. Special concern was given to how the two legal codends, the D25 and D40 codend, would perform under the commercial fishing condition. Considering the MLS regulation, the results from selection curves and exploitation pattern indicators indicated that the D25 and D40 codend would not perform satisfactorily in the fishing grounds where juvenile white croaker are relatively abundant. This was because the retention rate for white croaker with a length of 15.0 cm (the MLS) was 100% for the two codends, and the mean discard ratio (*dnRatio*) was 97.65% and 96.89% for the D25 and D40 codend, respectively. The implications of all these were that a substantial proportion of juvenile white croaker would be retained when they exist in the fishing grounds. Our study showed that the size selection of tested codends for white croaker would be improved by increasing the mesh sizes. For instance, by increasing mesh size to 45 mm (the D45 codend) a significant larger L50 value would be obtained.

Our results showed that the overall selective properties of all diamond mesh codends tested for white croaker were relatively poor. More research should be done to improve size selection of fishing gears for white croaker, especially for the juvenile fish, because high retention of undersized fish may have negative impact on the fisheries and the ecosystem of the study area. Taking into consideration the huge number of trawlers in the SCS (the reported figure of trawl fishing vessels were more than 7000 in 2018 [2]), it can be suggested that the bycatch situation of undersized white croaker is a serious issue, even if all trawlers use the legal mesh sizes in the trawl codend. Thus, the high retention of juveniles by this gear might have impacted the recruitment level of the species and be a reason for the overexploitation of the species. The ecosystem of the studied area might also be affected since juvenile white croaker is one of main prey of some economically important fish species, such as largehead hairtail (*Trichiurus lepturus*) [25]. Removing prey species would have an effect to the balances of food wed. Moreover, as white croaker is also one of the main target species of gillnet fishery [26], poor selection of trawl might affect the potential benefit of the fishermen targeting this species in the related fishery.

Some precaution is required in our study because the sea trial was based on finite number of hauls for each codend and the number of white croaker was limited to 677 individuals. Additionally, most of these fish were undersized, which would raise uncertainty in the estimation of the exploitation pattern indicators. Although catching juvenile fish was not the initial intention of our study, high proportion of undersized white croaker offered us excellent data to focus on the size selectivity of juvenile fish. With the consideration of poor size selectivity of codends used, action should be taken to protect juvenile fish resource, especially in the studied area where undersized white croaker is abundant and could be considered as a nursery place for the specific species.

Tokai et al. [27] investigated the size selection of white croaker using a diamond-mesh codend of 54 mm mesh size and survey data from several research vessels in the East China Sea. According to their results, the selective parameters, L50 and SR, of white croaker were 11.32 (CI: 10.86–11.78) cm, and 3.55 (CI: 2.81–4.29) cm, respectively. The L50 and SR of the D54 codend from our experiments were very close to the above-mentioned values. Despite the fact that confidence intervals of our results were relatively larger, they overlap with those found by Tokai et al. [27]. Considering the potential variation in fishing gear, season, ground, vessel, and population between the two experiments, the similarity in the results demonstrate the accuracy of our fishing experiments and data estimation.

It has been demonstrated that the accuracy of selectivity studies for fishing gears would be affected by the number of fish caught and length measured in the sea trials [28, 29]. The more fish is length measured, the less uncertainty will be obtained in the estimation of selective parameters. Herrmann et al. [28] stated that the covered codend method is a preferable choice when there might be not a high quantity of fish to be caught and length measured. Be aware of these outstanding literatures, and considering that the fisheries resources have been overexploited (in that situation it might be hard to have a large number of specimen), we chose the covered codend method in the sea trial. Though a finite number of fish, 677 individuals in total, was presented in our experiments, the uncertainties were taken into account by the double bootstrap in the data analysis. The confidence intervals of the selection parameters, L50 and SR, of the tested gears were at an acceptable level. The estimation of exploitation pattern indicators, however, was somehow affected by the spare number of fish above the MLS.

Although size selection of diamond-mesh codend for many important fishery species have been reported all around the world [30–33], the size selection for white croaker of the trawl fishery in SCS is firstly presented by this study. Previous studies have demonstrated that there are many factors affecting size selection of fish, such as mesh size, mesh shape, fish size, fish shape and environmental factors [12, 33, 34]. In our study, we simply focused on the mesh size. One reason for the poor size selection of the two legal codends can be related to the common characteristic of the diamond-mesh configuration in our experiments. Other designs, such as reducing the mesh number of the codend circumference, and using of a T90 (diamond-mesh turned 90°) or a square mesh codend could be proved more efficient as alternative options.

In conclusion, the results of this study show that 25 mm and 40 mm diamond mesh codends, currently used in the MMS regulations, all had high retention probability for juvenile white croaker. By increasing the mesh size of the codends, the size selection can be improved and retention of juvenile fish can decrease; however, without achieving a sustainable level for the stock at least for the tested mesh sizes. Future research work should focus on alternative codend designs mentioned above and in other fishing grounds of white croaker in the SCS.

## Supporting information

S1 Table Catch data for individual hauls. The catch data consist of towing time, depth, and the number of the studied species caught in the tested codend and cover and the subsampling ratio in each individual haul for each test.
(DOCX)
