# Supplementary material for: Effects of six codend meshes on the size selection of juvenile white croaker (Pennahia argentata) in demersal trawl fishery of the South China Sea
Source: PLoS One. 2021 Jul 16;16(7):e0253723. doi: 10.1371/journal.pone.0253723 (PMC8284606; doi:10.1371/journal.pone.0253723)
Supplement: S1 Table — The catch data consist of towing time, depth, and the number of the studied species caught in the tested codend and cover and the subsampling ratio in each individual haul for each test. (DOCX) [file pone.0253723.s001.docx]

S1 Table. Catch data for individual hauls. Haul number, Towing time (min), depth (m), and the number of fish for length measurement from the codend (*nR*), and cover (*nE*), while *qR* represents the sampling ratio of individuals in the codend, and *qE* represents the sampling ratio of individuals in the cover.

| codend | haul No | Tow time(min) | Depth (m) | *nR* | *qR* | *nE* | *qE* |
| --- | --- | --- | --- | --- | --- | --- | --- |
| D25 | 1 | 124 | 18 | 2 | 0.33 | 1 | 1.00 |
| D25 | 2 | 119 | 19 | 2 | 0.33 | 51 | 1.00 |
| D25 | 3 | 118 | 19 | 2 | 0.50 | 0 | 1.00 |
| D25 | 4 | 130 | 18 | 1 | 0.50 | 0 | 1.00 |
| D25 | 5 | 156 | 18 | 9 | 0.50 | 1 | 1.00 |
| D25 | 6 | 127 | 17 | 0 | 0.50 | 6 | 1.00 |
| D25 | 8 | 140 | 15 | 2 | 0.50 | 15 | 1.00 |
| D30 | 1 | 124 | 18 | 4 | 0.33 | 18 | 1.00 |
| D30 | 2 | 119 | 19 | 4 | 0.33 | 22 | 1.00 |
| D30 | 3 | 118 | 19 | 6 | 0.50 | 56 | 1.00 |
| D30 | 4 | 130 | 18 | 4 | 0.50 | 11 | 1.00 |
| D30 | 5 | 156 | 18 | 6 | 0.50 | 18 | 1.00 |
| D30 | 6 | 127 | 17 | 4 | 0.50 | 9 | 1.00 |
| D30 | 7 | 130 | 14 | 0 | 0.50 | 12 | 1.00 |
| D30 | 8 | 140 | 15 | 1 | 0.50 | 0 | 1.00 |
| D35 | 1 | 128 | 17 | 4 | 0.50 | 7 | 1.00 |
| D35 | 2 | 135 | 17 | 11 | 0.50 | 5 | 1.00 |
| D35 | 3 | 149 | 17 | 1 | 0.50 | 34 | 0.50 |
| D35 | 4 | 153 | 16 | 7 | 0.50 | 5 | 0.50 |
| D35 | 6 | 154 | 12 | 0 | 0.50 | 9 | 0.50 |
| D35 | 7 | 134 | 13 | 12 | 0.33 | 1 | 0.33 |
| D35 | 8 | 130 | 15 | 5 | 0.50 | 3 | 1.00 |
| D35 | 9 | 122 | 17 | 0 | 0.50 | 7 | 0.50 |
| D40 | 1 | 128 | 17 | 4 | 0.50 | 16 | 1.00 |
| D40 | 2 | 135 | 17 | 4 | 0.50 | 13 | 1.00 |
| D40 | 3 | 149 | 17 | 14 | 0.50 | 1 | 0.50 |
| D40 | 4 | 153 | 16 | 11 | 0.50 | 13 | 0.50 |
| D40 | 5 | 143 | 12 | 0 | 0.50 | 24 | 0.33 |
| D40 | 7 | 134 | 13 | 7 | 0.50 | 10 | 0.25 |
| D40 | 8 | 130 | 15 | 6 | 0.50 | 0 | 0.50 |
| D40 | 9 | 122 | 17 | 0 | 0.50 | 5 | 0.33 |
| D45 | 1 | 125 | 13 | 0 | 0.50 | 1 | 0.33 |
| D45 | 2 | 128 | 12 | 1 | 0.50 | 2 | 0.33 |
| D45 | 3 | 120 | 13 | 4 | 0.50 | 16 | 0.25 |
| D45 | 5 | 122 | 17 | 5 | 0.50 | 16 | 0.33 |
| D45 | 6 | 124 | 17 | 6 | 0.50 | 1 | 0.33 |
| D45 | 7 | 122 | 17 | 36 | 1.00 | 3 | 0.33 |
| D45 | 8 | 124 | 24 | 3 | 1.00 | 3 | 0.33 |
| D54 | 1 | 125 | 13 | 0 | 0.50 | 2 | 0.25 |
| D54 | 3 | 120 | 13 | 6 | 0.50 | 6 | 0.20 |
| D54 | 4 | 122 | 13 | 3 | 1.00 | 9 | 0.20 |
| D54 | 5 | 122 | 17 | 16 | 1.00 | 5 | 0.33 |
| D54 | 6 | 124 | 17 | 5 | 0.50 | 0 | 0.33 |
| D54 | 7 | 122 | 17 | 1 | 1.00 | 10 | 0.25 |
| D54 | 8 | 124 | 24 | 5 | 1.00 | 6 | 0.25 |
